# Medical Schools and Medical Protection

**Published:** 1915-06-05

**Authors:** 


### MEDICAL SCHOOLS AND MEDICAL PROTECTION.

### "The Hospital's" Criticism Endorsed.

The annual meeting of members of the London and Counties Medical Protection Society, Limited, was held at the offices, Craven Street, Strand, on May 26, under the presidency of Dr. G. Allan Heron. In the course of his address he endorsed The Hospital's criticism of the teachers at medical schools for not warning students of the risks inseparable from medical practice, and the need for joining a Medical Protection Society.

In proposing the reception of the annual report, the President briefly outlined the objects of the Society and its method of procedure when members sought its aid. Its aim was to defend medical practitioners who were unjustly attacked, using the term "practitioner" to include physicians, surgeons, and dentists. An applicant who desired to enlist the Society's aid made a statement, either verbally or in writing, to the General Secretary, who submitted a report of the case to the Council. The Council alone decided, after looking at the case as medical men and as men of the world, whether or not they would defend it. Legal points were referred to the Society's solicitors. No expression of opinion by any member of Council or by any official bound the Society to a course of action unless it had the authority of the Council as a whole; and that fact was required to be stated in any communication on a case. He emphasised that point because it had been a subject of misunderstanding. Any medical practitioner was liable at any time to find himself the subject of adverse and sometimes malignant criticism, no matter how carefully he ordered his conduct; and while no sensible man would take notice of gossip or tittle-tattle, it would be foolish of him to allow aspersions on his honour and integrity to go unchallenged. Even men of distinction were far from immune from such attacks, and had been glad of the Society's intervention. With regard to the liability of any medical man to be the subject of such attacks, he had had his attention directed to the article in The Hospital of May 22, in which that fact was emphasised.

The report, with the financial statement, was duly received, and the President then proposed its adoption. In doing so he said it had often struck him as remarkable that a greater proportion of medical men had not attached themselves to some protection society. There were numerous cases in which, when trouble came upon them, members of the profession expressed their great regret that they had not taken this precaution, but he, the President, could not avoid the feeling that they largely deserved the disappointment. The obstacle could not have been the membership fee, because that was within the competency of practically all. If the medical journals would put this aspect forcibly before their readers perhaps the next annual meeting would see a gratifying increase of membership.

As The Hospital, in the article to which he referred, said, the teachers at medical schools were very much to blame. A young man entering the profession did not realise the risks which were inseparable from the practice of medicine, no matter how careful he might be. He knew, from his medical friends, that some of the teachers did place the matter before their students and urge them to take steps for their protection by joining such a Society as this; but he believed the majority of the teachers did nothing of the kind. Dr. Heron then indicated the kind of cases with which the Society had recently dealt, and commented on the fact that in some cases, though the fees of counsel and solicitors were paid by the opposing party, when it came to the Society receiving its costs there was said to be nothing available, or the party went into bankruptcy; the Society seldom received anything like its just costs. Yet the party did not plead *in forma pauperis*. He thought these cases should be inquired into by the Law Society, or other authority. The balance-sheet disclosed a very satisfactory condition.

In conclusion he referred to an article in *The Medical Press and Circular*, urging amalgamation of the Society with the Medical Defence Union. He was in favour of such a combination, but prolonged efforts in this direction in 1896 did not bring any tangible result, and he did not think the present time was opportune for bringing the matter forward again.

The report and financial statement were unanimously accepted.

The President and various officers were re-elected, as were also the members of Council who retired by rotation—namely, Dr. Dempster, Dr. Fowler, Dr. Guthrie, and Mr. Sprawson.

